# Provings of Syphilinum

**Published:** 1890-07

**Authors:** Samuel Swan

**Affiliations:** New York


### PROVINGS OF SYPHILINUM.

SAMUEL SWAN, M. D., NEW YORK.

(1.) Jan. 27th.—Dr. H. took, at 3.30 P. M., a powder of one of Dr. Swan's high potencies of *Syphilinum;* another dose at 4 P. M., and at 4.35 P. M.

Had been suffering from a bad cold, with considerable cough and expectoration and catarrh, but that is now relieved very much by *Pulsat.*, some four or five days ago. The mouth, jaws, teeth, gums, and articulation of lower jaws have annoyed him by a soreness and tenderness for several days, so at times chewing food is uncomfortable. The scaly eruption over *os coxæ* is again present, and has annoyed him for several weeks; at present it is worse than for some time.

At 4.40 P.M. (five minutes after the third dose) noticed aching, as of bruise, in adductor muscles of left leg. At 4.45 P. M., the pain had extended to anterior tibial region, lower third; also similar pain in right costal region anteriorly. At 5 P. M., took fourth powder. The pain in costal region continues slightly, and slight dull frontal headache is noticeable. All of the pains thus far are of a bruised character.

Jan. 28th.—No other symptoms were noticed until this morning. He got up rather hurriedly, with colicky pains in abdomen, and rather loose diarrhœaic stool, at 7 A. M. Had a similar attack at 9 A. M., with some tenderness of anus after stool. Noticed the nasal discharge to be slightly discolored with blood on two different occasions this morning; had not noticed this before. Noticed also the cough has almost entirely, if not quite, gone.

Jan. 29th.—No further symptoms noted. The cough has not troubled him, but the nose still annoys him at times. The jaws still ache when chewing.

(2.) October 17th, 1888.—Dr. J. reports the following case of poisoning: In early part of February, 1887, I attended to a cut on head, and after binding it up, tried to tear the bandage. It tore hard, and when it gave way my left thumb-nail cut the second joint of right second finger. I paid no attention to it, as all former cuts had healed nicely. Next day was called to a case of prolapsus uteri, and replaced it. Since learned that the woman had been a prostitute, and had syphilis. The next night was called to a case of labor; the woman has since died, and I have reason to think of syphilis in some form. Not then knowing these facts, and my finger not healing, wondered at it. Some time in May my skin became covered with the syphilitic maculæ, and then I began to realize what was the trouble, and placed myself under the care of a Hahnemannian physician.

After the maculæ followed the headache; mind dwelt upon suicide; had to exert all my will to keep from it. The sufferings were terrible; no sleep, except with cold water or alcohol on a cloth, and laid upon eyes and forehead and temples; must have head high. Was in the country when at the worst (June, 1887). One day feeling so very despondent, took *Aurum*$^{cm}$ on tongue, and slept all that night, and continued to improve, all except the hearing. Began to grow deaf, with all the noises possible (still have the hissing); could not hear a person without they halloed very hard; no sound from clocks, or trains passing; and one day, when I went down-town, it was as still as death. Hearing is nearly normal now. Kept along feeling fairly, until May 8th, 1888, when a severe chill took me, high fever; could not lie, sit, or stand; better moving, though that was painful. Took *Rhus-tox*$^{cm}$ at last, which helped; better next day. Then began to grow sick; swelling of right epididymis, which formed an abscess, and discharged. Strabismus of left eye, with diplopia; iritis. Headaches slight; mind good, except disheartened. Dyspepsia, which *Mercury* aggravated. Now began to eat pop-corn, and have continued to-day to eat two quarts after dinner. In June, 1887, lost my eyebrows, which dropped out, and in June, 1888, my countenance was awful; weight went down to about 135 pounds, now weigh 165. The sore on finger was eight months healing, and still shows redness, and is less firmly adherent to bone.

To-day feel better than for two years, although there is still the noise in both ears, and difficulty in hearing some noises, such as front-door bell, ticking of my watch, which I have to press against ear, and some words which begin with S, T, and a few other letters. Have begun to use glasses to read and write with.

(3.) *Syphilinum* in high potency has cured tinnitus aurum, especially in right ear, worse at night.

